# Low latency iterative reconstruction of first pass stress cardiac perfusion with physiological stress using graphical processing unit

**DOI:** 10.1186/1532-429X-15-S1-E10

**Published:** 2013-01-30

**Authors:** Sébastien Roujol, Tamer A Basha, Christophe Schülke, Martin Buehrer, Warren J Manning, Reza Nezafat

**Affiliations:** 1 Medecine, BIDMC / Harvard Medical School, Boston, MA, USA; 2 Radiology, Beth Israel Deaconess Medical Center and Harvard Medical School, Boston, MA, USA; 3 Institute for Biomedical Engineering, ETH Zurich, Zurich, Switzerland

## Background

Cardiac MR perfusion has been shown to provide high diagnostic accuracy in detection of the coronary artery disease [1]. We have recently installed an MR-compatible supine bicycle mounted on the scanner table, which allows performing CMR perfusion immediately after physiologic stress. However, patients are unable to sustain a breathold after physical exercise, limiting the choice of acceleration techniques such as k-t approaches. Additionally, due to subject motion during exercise, coil sensitivity map are inaccurate resulting in imaging artifacts in conventional parallel imaging reconstruction. Compressed sensing (CS) is an alternative acceleration technique that enables high acceleration even without exploiting temporal dimension or need for coil maps. However, iterative CS reconstruction of randomly undersampled k-space is lengthy, performed off-line and is not usually integrated into the workflow of a clinical scan which requires viewing and initial assessment on the scanner console and storing the clinical images on the hospital PACS system. In this proposal, we aim to develop an accelerated iterative CS reconstruction workflow for reconstruction of CS acquired perfusion data using physical stress perfusion. 

## Methods

**Figure 1:**
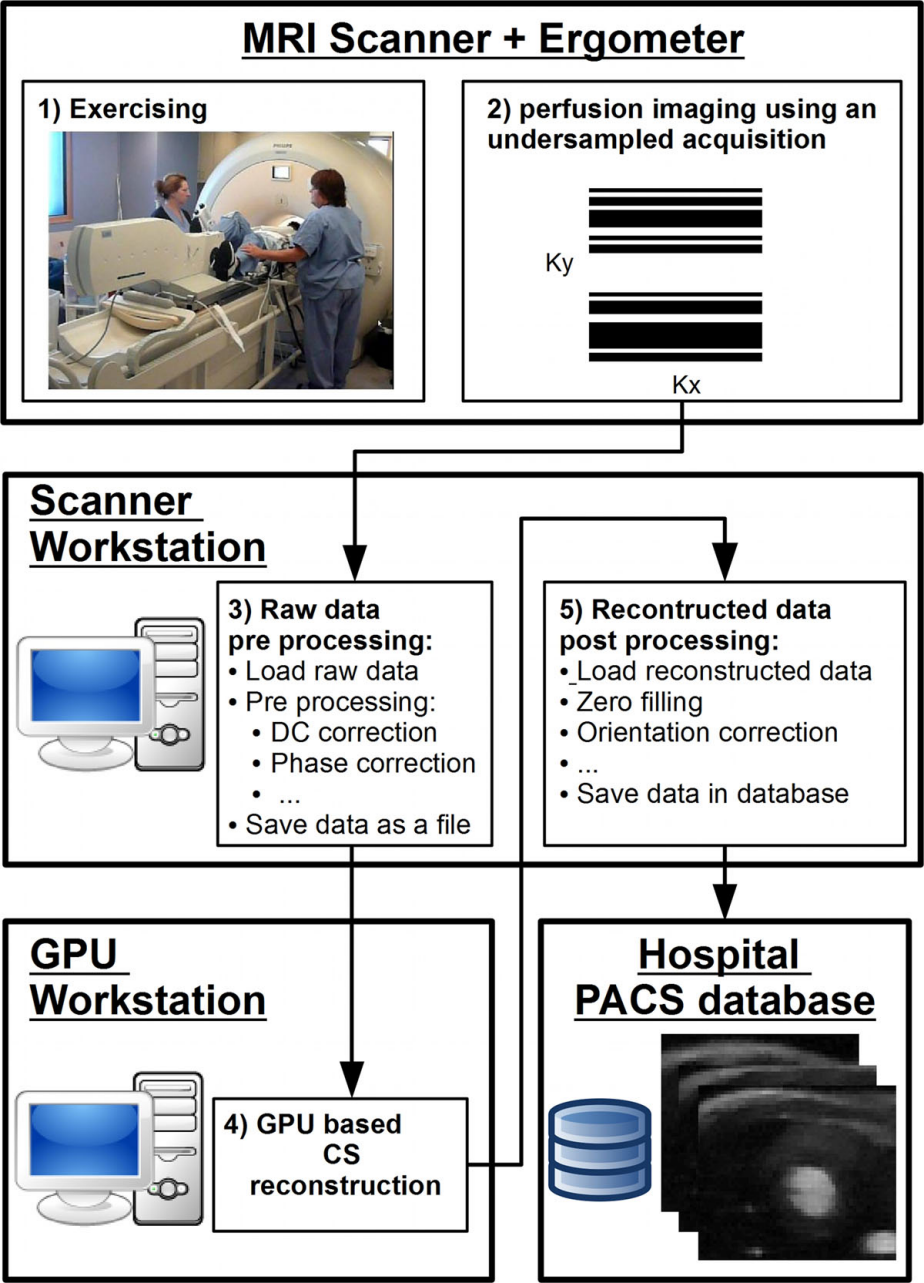
Workflow of the iterative CS reconstruction for CMR perfusion immediately after supine bicycle physiologic stress using a remote workstation equipped with GPU. The undersampled k-space data are sent to the GPU workstation using hospital network. After reconstruction, the data are sent back to the scanner workstation for final post-processing using the ReconFrame platform for viewing and storage in clinical hospital PACS system and further analysis of images. This allows integration of CS-accelerated CMR perfusion into the clinical workflow with minimal user interaction.

Figure 1 shows the workflow of the accelerated CMR perfusion reconstruction. After completion of the CMR perfusion sequence, the reconstruction process is manually started by CMR technologist using an in-house graphical user interface. All the subsequent reconstruction steps are then performed automatically without any user interaction. The raw data are pre-processed on the scanner workstation and sent to a dedicated computer for reconstruction (equipped with graphic processing unit (GPU) NVIDIA Tesla) and finally sent back to the scanner workstation and the PACS database. Pre- and post-processing are performed using the ReconFrame platform (Gyrotools, Zurich, Switzerland). The GPU-based CS reconstruction is implemented using a fast alternating minimization approach [2]. Since this reconstruction is iterative and voxel-independent for each iteration, the parallelization level of the GPU implementation was set to the voxel level. The presented workflow has been tested during bicycle ergometer stress CMR perfusion exams in healthy subjects using a 1.5T Philips scanner and a prospective 4× CS-accelerated CMR perfusion sequence. Computation time and latency of each reconstruction step was measured and compared to a non-parallelized implementation.

## Results

The proposed workflow allows reconstruction and viewing of the CS accelerated perfusion on the scanner console. The GPU-based implementation provides a 12-fold reduction in reconstruction time with an overall latency of 5 min 15s (Table 1).

**Table 1:** Computation time of the different reconstruction steps obtained for a complete CMR perfusion dataset.

Load raw data on the scanner	10s
Pre-process raw data on the scanner	15s
Save pre-processed raw data on the scanner	25s
Send data to the GPU workstation	14s
Compute the CS reconstruction	4 min, 5s (51 min)
Transfer the data back to the scanner workstation	1s
Post-process the reconstructed data and save to the scanner database	5s
Total	5 min, 15s (52 min, 10s)

The reported times correspond to the treatment of the complete perfusion dataset (32 channels, 90 dynamics, 3 slices, image size=132x132, 100 iterations) for each reconstruction step. Computation time obtained with the CPU only implementation is also reported (brackets). The proposed GPU reconstruction provides an acceleration factor > 12 compared to the CPU only implementation and provides an overall reconstruction time with a significantly reduced latency.

## Conclusions

GPU based CS-reconstruction significantly improved the reconstruction time and guarantee a minimal latency required for optimized clinical MR protocol for CMR perfusion during physical stress.

## Funding

NIH:R01EB008743-01A2
